# Universal substance use care for adolescents with chronic medical conditions: a protocol to examine equitable implementation determinants and strategies for SBIRT at a pediatric hospital

**DOI:** 10.1186/s13722-024-00492-4

**Published:** 2024-09-11

**Authors:** Faith Summersett Williams, Robert Garofalo, Niranjan S. Karnik, Geri Donenberg, Hayley Centola, Sara Becker, Sarah Welch, Lisa Kuhns

**Affiliations:** 1 Department of Pediatrics, Feinberg School of Medicine, The Potocsnak Family Division of Adolescent and Young Adult Medicine, Northwestern University, Ann & Robert H. Lurie Children’s Hospital, Chicago, IL USA; 2 Institute for Juvenile Research, Department of Psychiatry, College of Medicine, Institute for Juvenile Research, University of Illinois at Chicago, Chicago, IL USA; 3 Department of Medicine, Center for Dissemination and Implementation Science, University of Illinois at Chicago, Chicago, IL USA; 4 Department of Pediatrics, Michigan Medicine, Ann Arbor, MI USA; 5 Department of Psychiatry, Feinberg School of Medicine, The Center for Dissemination and Implementation Science (CDIS), Northwestern University, Chicago, IL USA; 6 Department of Emergency Medicine, Feinberg School of Medicine, Northwestern University, Chicago, IL USA

**Keywords:** Health equity, Implementation science, Substance use & misuse, Chronic medical conditions (CMCs), Pediatric hospital, Inpatient hospitalization

## Abstract

**Background:**

Adolescents with chronic medical conditions (CMC) use alcohol and marijuana at levels equal to or even greater than their peers without CMC and are more likely to initiate substance use at 14 years or younger. Approximately 33% of adolescents with CMC binge drink alcohol and 20% use marijuana. When using substances, adolescents with CMC are at elevated risk for problem use and adverse consequences given their medical conditions. Although there has recently been progress integrating substance use services into adult hospitals, there has been almost no implementation of standardized substance use services into pediatric hospitals for adolescents with CMC. Screening, Brief Intervention, and Referral to Treatment (SBIRT) for adolescents is an evidence-based, public health approach to promote the early detection and intervention of risky alcohol use in high-risk youth. This paper describes a study protocol combining two leading implementation science frameworks, the Consolidated Framework for Implementation Research (CFIR) and the Health Equity Implementation framework (HEIF), to engage pediatric hospital partners (hospital staff and clinicians, patients with CMC, and caregivers) to identify and specify contextual determinants of SBIRT implementation, which can be used to derive implementation strategies to optimize SBIRT adoption, reach, and fidelity.

**Method:**

This study will use semi-structured interviews and focus groups with pediatric hospital partners (e.g., hospital staff and clinicians, adolescent patients, and caregivers) to identify SBIRT implementation determinants, using semi-structured interview and focus group guides that integrate CFIR and HEIF dimensions.

**Discussion:**

Understanding implementation determinants is one of the first steps in the implementation science process. The use of two determinant frameworks highlighting a comprehensive set of determinants including health equity and justice will enable identification of barriers and facilitators that will then map on to strategies that address these factors. This study will serve as an essential precursor to further work evaluating the feasibility of and the degree of engagement with SBIRT among this vulnerable pediatric population.

## Background

In the United States one-in-four adolescents is growing up with a chronic disease [1]. To stay healthy, youth with chronic medical conditions (CMC) often require frequent medical appointments, prescription medication to control their condition, and periodic laboratory tests [2]. Youth with CMC are often instructed to avoid activities and behaviors that can compromise their health while navigating the typical physical, mental, and emotional challenges of adolescence [3, 4]. Yet an emerging literature on the intersection of substance use (SU) and chronic illness suggests that despite their medical vulnerability, youth with CMC experience substance use-related consequences at levels greater than their peers without CMC [5, 6]. Approximately 13% of adolescents with chronic medical conditions (CMC) binge drink alcohol and 20% use marijuana [7]. Although similar percentages of youth with CMC and youth without CMC experiment with substances in early adolescence, youth with CMC are more likely to initiate substance use at 14 years or younger. Moreover, youth with CMC are disproportionately likely to develop a SU disorder by older adolescence and young adulthood [5, 7]. For all youth, SU poses risks for acute harm from accidents and injuries [8]; for youth with CMC, SU poses additional risks from adverse medication interactions, increased treatment non-adherence, and poor disease control [7, 9, 10]. Undetected and untreated SU in this vulnerable population can have far-reaching consequences beyond the individual patient, placing significant stress on families, communities, and healthcare systems [11].

Despite the public health concerns related to SU among adolescents with CMC, visits in subspecialty ambulatory or inpatient care rarely include screening or guidance regarding SU missing a key opportunity. Likewise, the increased risks among youth with CMC underscore the importance of integrating SU services in pediatric hospitals where many adolescents with CMC interface with the healthcare system. Screening, Brief Intervention, and Referral to Treatment (SBIRT) for adolescents is an evidence-based, public health approach that is ideally suited for the pediatric hospital setting. ***S****creening* quickly assesses the severity of SU and determines the appropriate level of treatment using evidence-based tools. ***B****rief****I****ntervention* focuses on increasing adolescents’ insight and awareness regarding SU and motivation toward behavioral change. ***R****eferral to****T****reatment* provides adolescents identified as needing treatment with information about organizations providing treatment specifically for SU. Advantages of SBIRT include quick and efficient screening, identification of undetected cases or cases early in the trajectory of misuse, time-limited intervention, and facilitation of co-located treatment [12].

The Substance Abuse and Mental Health Services Administration (SAMHSA) recommends that health care clinicians use SBIRT during routine health service visits given SBIRT’s cost-effectiveness, efficiency, and ease of integration into a range of settings beyond SU specific services [13]. Consistent with these recommendations, pediatric outpatient primary care and emergency departments [12] have successfully integrated SBIRT to evaluate SU [14–16]. To our knowledge a systems-level approach has never before been applied to implement SBIRT in inpatient units in a pediatric hospital. Although implementing SBIRT in an inpatient setting with patients who have CMCs may pose additional implementation challenges we assert that implementation studies are needed because this oversight may disproportionately harm youth with CMC who are significantly more likely to require an inpatient stay [17].

Translating evidence-based interventions into routine general practice in healthcare has historically been a slow, challenging process [18]. As noted by Moullin and colleagues, the appropriate use of implementation frameworks prior to and throughout an implementation effort can guide the design and execution of trials, inform the theoretical and empirical thinking of transdisciplinary research teams, and assist in the interpretation of results [19]. The Consolidated Framework for Implementation Research (CFIR) is a widely used conceptual framework of 39 constructs organized across 5 domains that can be used to theorize determinants (barriers, facilitators, constraints) of implementation across diverse settings [20]. Moreover, CFIR provides a structure to systematically assess the context where implementation occurs [21]. The application of CFIR to identify implementation determinants ideally is inclusive of the perspectives and experiences of *all* partners, which in the case of adolescents with CMC would include pediatric hospital staff, clinicians, patients, and caregivers [20, 22, 23]. A key critique of CFIR is that it does not sufficiently account for systemic and structural determinants that perpetuate inequities along the healthcare continuum. CFIR’s deployment as a presumptively discrimination-free tool may obscure the influences of oppression-related factors on the implementation process, thereby limiting our understanding of barriers and facilitators that perpetuate health disparities. Social justice theories suggest that when consideration of structural inequities is not at the forefront of analyses, our ability to identify social factors that determine study outcomes is limited [24, 25].

Oppression in the form of isms and phobias such as racism, sexism, classism, homophobia, and xenophobia are fundamental aspects of socialization that shape institutions [26]. These socialization processes provide advantages and opportunities to groups with a closer proximity to power than others. In the context of healthcare, structural oppression operates within and across interconnected systems that are adaptive in shaping and reinforcing both health inequities and the research to practice gap [27]. Ignoring the role and impact of these structural forces in the context of implementation and implementation science can lead to inaccurate explanations as to why inequities exist and sub-optimal interventions that perpetuate health inequities.

Incorporating health equity domains within implementation frameworks like CFIR may optimize the scientific yield and equity of implementation efforts by assessing and addressing implementation and equity barriers simultaneously [28]. The Health Equity Implementation Framework (HEIF) posits determinants that predict successful and equitable implementation outcomes within healthcare and clinical practice settings [29]. Within each domain in the HEIF there are several determinants or specific factors that are measurable and, together in constellation with other determinants, clarify barriers, facilitators, moderators, or mediators to implementation and health equity success [28]. We will use the HEIF to establish theory-driven factors that predict SBIRT implementation and equity success within three health equity domains: cultural relevance (e.g., inclusive language), clinical encounters (e.g., patient-provider interactions), and societal context (e.g., sociopolitical forces) for this vulnerable pediatric population and the inpatient setting [30]. The current protocol intentionally combines CFIR and HEIF to guide comprehensive identification of key determinants (barriers, facilitators) of equitable SBIRT implementation in the inpatient setting in a large urban pediatric hospital in the Midwest for adolescents with CMC.

The purpose of this study is to engage pediatric hospital partners (hospital staff and clinicians, patients with CMC, and caregivers) to identify and specify contextual determinants of SBIRT implementation, which can be used to derive implementation strategies to optimize SBIRT adoption, reach, and fidelity.

## Methods/design

### Design

This study will conduct semi-structured interviews and focus groups with pediatric hospital partners (e.g., hospital staff and clinicians, adolescent patients, and caregivers) to identify SBIRT implementation determinants, using pre-set guides that integrate CFIR and HEIF dimensions. Individual interviews with hospital staff (i.e., nurses, trainees, physicians, psychologists, social workers, and administrators from information systems) will focus on five CFIR domains to identify barriers and facilitators of implementation (i.e., intervention characteristics, inner setting, outer setting, and the characteristics of individuals involved in the implementation process) [22]. Two separate focus groups with patients and caregivers will address the same five CFIR domains with an additional three HEIF domains (i.e., culturally relevant factors, clinical encounter or the patient-provider interaction, and societal context) [28]. Taken together, these discussions will identify and prioritize determinants of SBIRT implementation and necessary adaptations to the intervention.

### Setting

The pediatric medical center site is located in an urban area in the Midwest and has a longstanding history of serving children and adolescents with CMC. The pediatric hospital offers expert care by a multidisciplinary team and tailored to children and adolescents’ unique needs. The pediatric inpatient unit has an infrastructure on which to build SBIRT’s implementation and includes a social work consultation service and a substance use outpatient treatment program which can serve as referral sources for treatment upon discharge.

### Recruitment

We will employ purposive and convenience sampling to recruit a mix of staff and clinicians, patients with CMC, and caregivers of patients. We will identify 25 hospital staff and cliniciansfrom 5 divisions (e.g., Hematology, Oncology, Neuro-Oncology, & Stem Cell Transplantation; Pulmonary and Sleep Medicine; Transplantation; Emergency Medicine; and General Medicine) who meet the following inclusion criteria: (1) aged 18 years or older; (2) employee or faculty at urban Midwestern pediatric hospital; and (3) position in the patient workflow, oversight of workflow or technical implementation of workflow in which SBIRT is likely to be imbedded. We will intentionally recruit at least 2 individuals from different disciplines, namely nurses, physicians, psychologists, social workers, and administrators from hospital information systems.

We will recruit 10 adolescent patients and 10 caregivers through clinician referral and self-referral using flyers on hospital and patient-facing listservs and newsletters. Adolescent inclusion criteria are: (1) ages 12–19; (2) able to speak/read English; (3) a diagnosed CMC (4) history of inpatient admission at the pediatric hospital; (5) willing and able to provide informed consent/assent; (6) parent or caregiver provides permission to participate (in English). Adolescents will be *excluded* if they are unable to provide informed consent due to severe mental or physical illness at the time of enrollment (based on clinician assessment). Parent/caregiver inclusion criteria include: (1) ages 18 and older; (2) able to speak/read English; (3) willing and able to provide informed consent/assent; (4) child has a diagnosed CMC; and (5) history of inpatient care at the pediatric hospital.

The study team will contact potentially eligible participants via telephone and/or email using an introductory script. Interested individuals will be scheduled to participate and consented/assented immediately prior to the interview or focus group. Youth under 18 years of age will provide written assent and their caregivers will provide written consent. Youth aged 18 or older will provide written consent to participate. Adolescents and caregivers will be able to enroll independently in the study (i.e., within or outside of dyads).

### Qualitative interview guide development

We will use semi-structured interview and focus group guides to identify and describe determinants of implementation of SBIRT in the pediatric inpatient setting. To explore the CFIR determinants, we will adapt the publicly available CFIR Universal Interview Guide to address the primary study questions. The CFIR guide has been widely used across a variety of populations and provides example qualitative interview questions based on CFIR determinants (Table 1) [31]. The interview will address all five CFIR domains: (1) intervention characteristics (e.g., compatibility of each element of SBIRT with usual care in the pediatric hospital setting), (2) partner characteristics (e.g., attitudes toward SBIRT, willingness to screen/be screened), (3) inner setting (e.g., compatibility of SBIRT with current medical services), (4) outer setting (e.g., policies and incentives to implement SBIRT, and (5) process (e.g., strategies to support SBIRT implementation) [20]. To explore HEIF determinants, we will use the publicly available HEIF interview guide (Table 2) [28] to address domains known to affect health disparities and equity: (1) culturally relevant factors, such as medical mistrust, demographics, or biases of recipients [32–35]; (2) clinical encounter or patient-provider interaction [36–38]; and (3) societal context including physical structures, economies, and social and political forces [39–41].

**Table 1:** Example SBIRT program qualitative interview questions using CFIR

CFIR Domain	Question	Probe
Intervention Characteristics	What are the primary goals and outcomes that your team would want to see from SBIRT?	Do you think these goals will be different or the same across the different departments and teams in the hospital? How can we align goals across stakeholder groups?
Inner Setting	How well does SBIRT fit with your values and norms and the values and norms within the organization?	Values relating to interacting with patients and families (e.g., shared-decision making vs. being more directive)?
Outer Setting	Can you tell me what you know about any other organizations that have implemented similar substance use services?	Does implementing SBIRT provide an advantage to your organization over others?
Individual Characteristics	How do you feel about using SBIRT in your setting?	How do you feel about using SBIRT-A in your department? Do you have any feelings of anticipation? Stress? Enthusiasm? Why?
Process	Are there people in your section or division who are likely to champion using SBIRT?	What position do these champions have in your organization? How do you think they will help with implementation? How do you think they will help with getting people to use SBIRT?

**Table 2:** Example SBIRT program qualitative interview questions using the HEIF

HEIF Domain	Question	Probe
Clinical Encounter (Patient-Provider Interaction)	How do your conversations about your health and treatment plan usually go with your provider?	Do you feel not understood by your doctor or nurse? Have you ever felt treated differently from others when getting treatment for [health problem]?
Cultural Factors of Recipients	Do teens and parents trust the hospital?	Do teens and parents trust the healthcare providers in the hospital? Do healthcare providers respect teens and parents’ thoughts and concerns about their treatment plans?
Societal Context	Do you feel there is a stigma against people, especially teens, who need help with drug and alcohol use?	Do you think these teens may be treated differently when getting help for drug and alcohol use?

### Qualitative interview and focus group delivery

Staff and clinician interviews will be conducted 1-on-1 via Zoom by a trained PhD-level behavioral scientist, audio-recorded, and last approximately 45–60 min. Separate adolescent and caregiver focus groups will occur in-person or via Zoom, last about 90 min, and use audio and graphic recording, i.e., translating the main themes and ideas discussed during the focus groups into a drawing. Adolescent patient and caregiver focus groups will be conducted by the principal investigator in groups of approximately 5–10 attendees. Audio recordings will be transcribed verbatim and graphic recording will be done in real-time. Graphic recording engages participants in real-time member-checking to validate understanding of responses. Participants will be asked to comment on or suggest corrections to the themes reflected in the graphic. The final drawing will be shared as a PNG file with participants via separate emails to preserve confidentiality. The drawing will exclude identifiers or information that reveal participants in the group. Data collection will stop or extend until data saturation is reached. Immediately following each interview or focus group, the interviewer and/or facilitators will write corresponding field notes indicating contextual details and nonverbal cues. Undergraduate-level research assistants will review 5 min of all audio transcripts for accuracy. We will not return transcripts to participants for review. Hospital staff, patients, and caregivers will receive $25 for their time and effort.

### Qualitative analysis

The transcribed and de-identified clinician and staff interviews and patient and caregiver focus groups will be analyzed for thematic content [42] using a deductive approach to identify which CFIR and HEIF framework determinants influence SBIRT program implementation [43]. The research team will develop a codebook by pulling determinants directly from CFIR and HEIF and including code definitions and application guidelines. Two trained coders with qualitative experience will independently review and rate each transcript using a modified RADaR method [44]. This method is acceptable for inductive and deductive analysis and has multiple advantages over qualitative software. Since our project is deductive, focused on identifying the relevance of identified facilitators and barriers to SBIRT implementation, this method allows us to spend less time coding and more time identifying, expanding, and linking themes. The RADaR method retains methodological rigor while reducing the costs associated with software training and burden. Using this method, the content from each transcript will be organized into a data table, codes applied, and inter-rater reliability assessed after the first two transcripts are coded. Upon completion, the coders will identify larger themes and corresponding subthemes to organize as a “thematic map” to illustrate potential thematic relationships. The two coders will then review the validity of and refine the thematic map by comparing it to interview transcripts. Once the thematic map is internally consistent and sufficiently describes the data, we will finalize the map’s major and minor themes and provide corresponding participant quotations that depict determinants of SBIRT implementation.

During the coding process, the coders will meet weekly and consult with team members with expertise in implementation science and SBIRT to identify themes and constructs to refine the thematic map. After the thematic map is finalized, the coders will enter the CFIR determinants into the “CFIR-Expert Recommendations for Implementation” matching tool. The tool generates specific implementation strategies as potential candidates to implement SBIRT in the pediatric hospital setting, which will lay the groundwork for future work [45].

## Discussion

This protocol describes an implementation design, with a focus on equity, of a pilot study seeking to prevent and intervene on SU among adolescents with CMC using SBIRT in inpatient units at a pediatric hospital. The study will extend the evidence base for SBIRT to adolescents with CMC who are susceptible to SU but have limited opportunity for intervention in traditional settings. The design has several strengths, including its focus on a population typically overlooked scientifically and clinically in the SU prevention and intervention literature and practice. In addition, this study provides a unique research opportunity to examine whether a comprehensive process of identifying implementation determinants focused on health equity can help to identify implementation strategies for use in the pediatric hospital setting.

Notably, a major limitation of implementation research has been a lack of studies testing the impact of health equity and justice on an implementation strategy and implementation effectiveness. This protocol is a critical precursor to addressing this significant gap by enabling the investigation of a broad range of implementation determinants, which will inform the selection of implementation strategies for later testing as potential mediators of implementation effectiveness.

Implementation disparities are rooted in social oppression, which includes structural determinants of health, and are exacerbated by multiple levels of influences. The domains included in the HEIF are not exhaustive but are one step towards integration of health equity into existing implementation determinant frameworks. Future research should focus on incorporating aspects of justice into implementation determinant, process, and evaluation frameworks which move away from an equity deficit-based approach (e.g., providing accommodations based on inequities) to a justice or liberation perspective (e.g., removing the structural or societal barriers that contribute to the inequities). Future research must also focus on identifying and testing implementation strategies that address health equity and justice determinants in pediatric healthcare systems.

The scientific significance of this research lies in its ability to provide universal and equitable SU treatment to a vulnerable pediatric population as well as advance implementation science by testing whether consideration of a broad range of equity-focused implementation determinants can inform the selection of implementation strategies relevant for the pediatric hospital setting.

## Data Availability

Not applicable.

## Abbreviations

CMC: Chronic Medical Condition(s)
SBIRT: Screening, Brief Intervention, and Referral to Treatment
CFIR: Consolidated Framework for Implementation Research
HEIF: Health Equity Implementation Framework
SU: Substance Use
EHR: Electronic Health Record
